# Supplementary figures and images for: Phylogenomics of Plant-Associated Botryosphaeriaceae Species
Source: Front Microbiol. 2021 Mar 18;12:652802. doi: 10.3389/fmicb.2021.652802 (PMC8012773; doi:10.3389/fmicb.2021.652802)

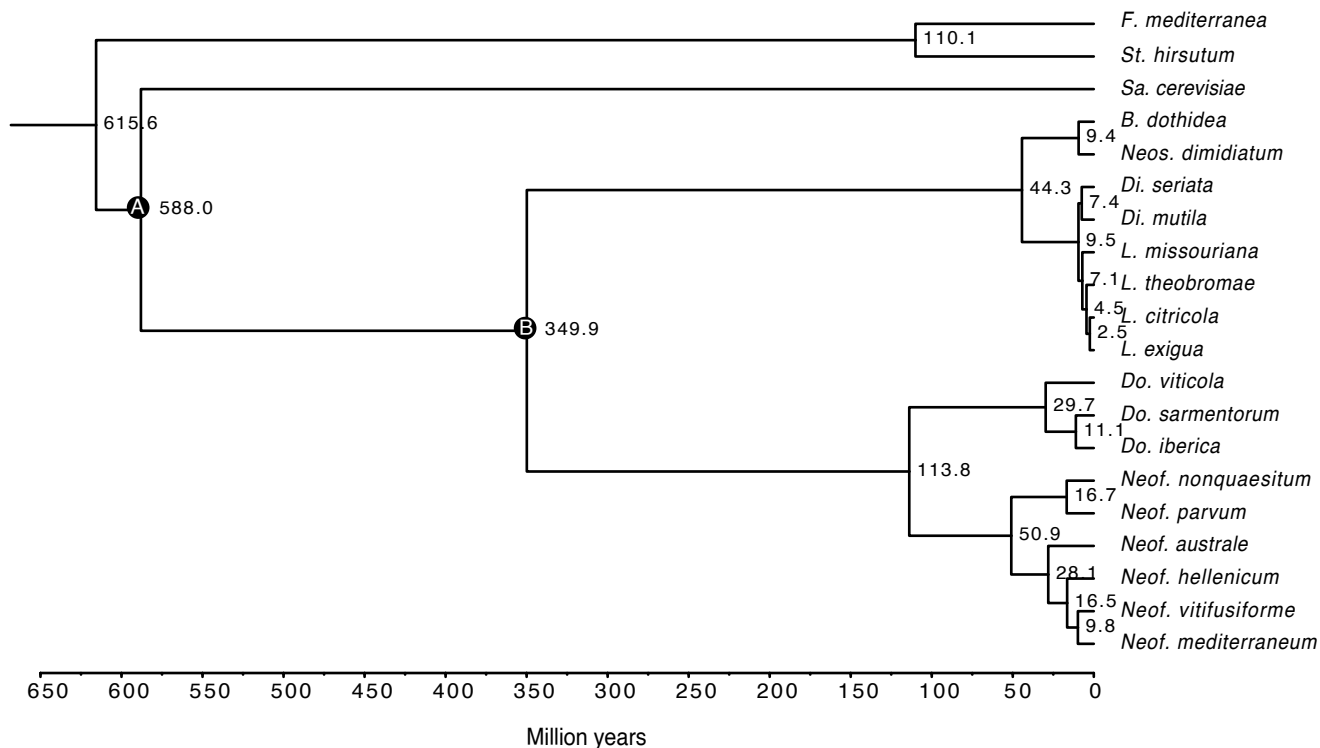

**Supplementary Figure 1.** Clock calibrated tree generated with BEAUti and BEAST.

Supplement: Supplementary file 1 [file Image_1.pdf]
