# Supplementary material for: Phylogenomics of Plant-Associated Botryosphaeriaceae Species
Source: Front Microbiol. 2021 Mar 18;12:652802. doi: 10.3389/fmicb.2021.652802 (PMC8012773; doi:10.3389/fmicb.2021.652802)
Supplement: Supplementary file 2 [file Image_2.pdf]

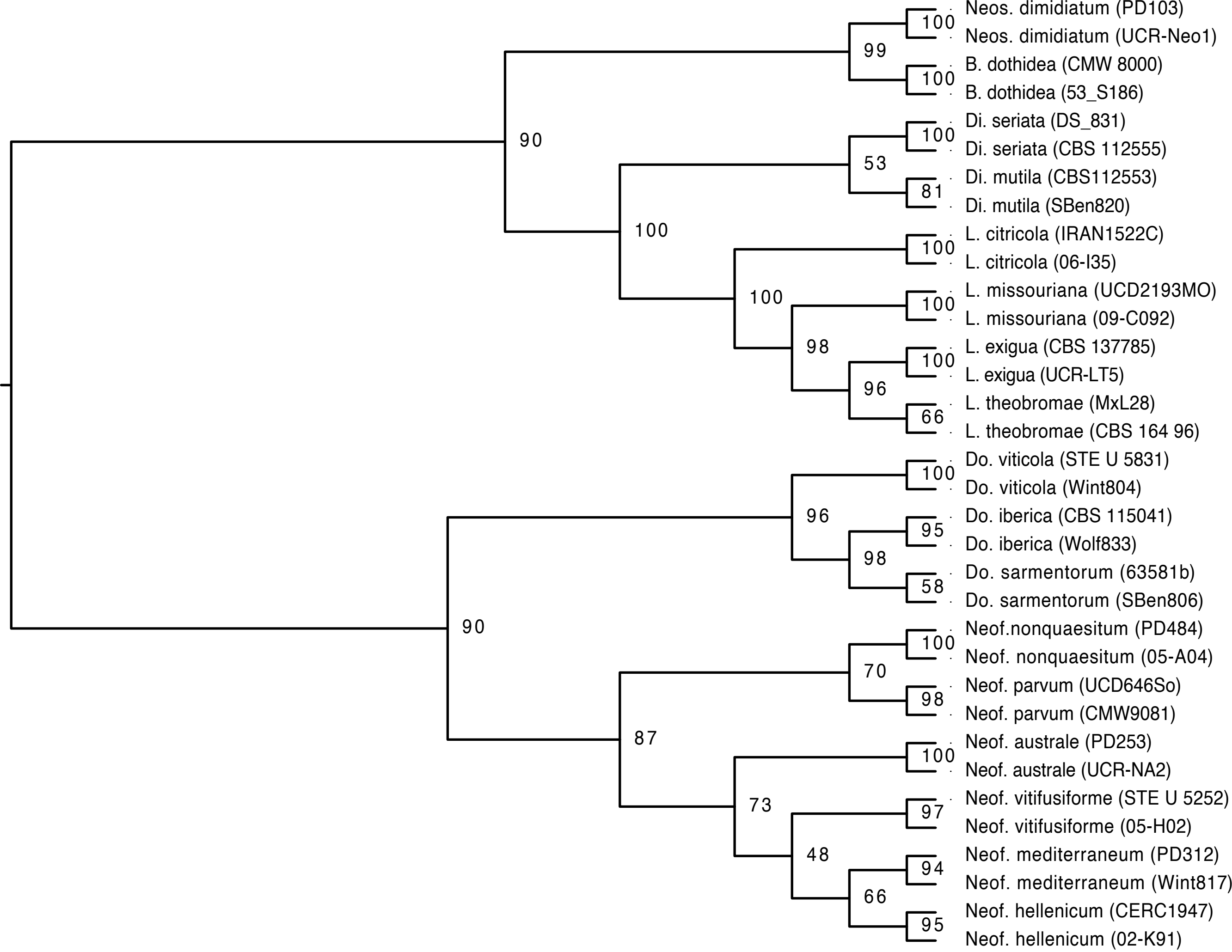

Supplementary Figure 2. Maximum-likelihood tree from ITS and TEF markers from different isolates of the Botryosphaeriaceae species in this study.
